# A genome-wide association study for blood lipid phenotypes in the Framingham Heart Study

**DOI:** 10.1186/1471-2350-8-S1-S17

**Published:** 2007-09-19

**Authors:** Sekar Kathiresan, Alisa K Manning, Serkalem Demissie, Ralph B D'Agostino, Aarti Surti, Candace Guiducci, Lauren Gianniny, Nöel P Burtt, Olle Melander, Marju Orho-Melander, Donna K Arnett, Gina M Peloso, Jose M Ordovas, L Adrienne Cupples

**Affiliations:** 1 National Heart Lung and Blood Institute's Framingham Heart Study, Framingham, MA, USA; 2 Broad Institute of Massachusetts Institute of Technology and Harvard University, Cambridge, MA, USA; 3 Cardiovascular Disease Prevention Center, Cardiology Division, Massachusetts General Hospital, Harvard Medical School, Boston, MA, USA; 4 School of Public Health, Boston University, Boston, MA, USA; 5 Department of Mathematics and Statistics, Boston University, Boston, MA, USA; 6 Department of Clinical Sciences, Hypertension and Cardiovascular Diseases, University Hospital Malmö, Lund University, Malmö, Sweden; 7 Diabetes and Endocrinology, University Hospital Malmö, Lund University, Malmö, Sweden; 8 Department of Epidemiology, University of Alabama at Birmingham, Birmingham, AL, USA; 9 Nutrition and Genomics Laboratory, Jean Mayer-USDA Human Nutrition Research Center at Tufts University, Boston, MA, USA

## Abstract

**Background:**

Blood lipid levels including low-density lipoprotein cholesterol (LDL-C), high-density lipoprotein cholesterol (HDL-C), and triglycerides (TG) are highly heritable. Genome-wide association is a promising approach to map genetic loci related to these heritable phenotypes.

**Methods:**

In 1087 Framingham Heart Study Offspring cohort participants (mean age 47 years, 52% women), we conducted genome-wide analyses (Affymetrix 100K GeneChip) for fasting blood lipid traits. Total cholesterol, HDL-C, and TG were measured by standard enzymatic methods and LDL-C was calculated using the Friedewald formula. The long-term averages of up to seven measurements of LDL-C, HDL-C, and TG over a ~30 year span were the primary phenotypes. We used generalized estimating equations (GEE), family-based association tests (FBAT) and variance components linkage to investigate the relationships between SNPs (on autosomes, with minor allele frequency ≥10%, genotypic call rate ≥80%, and Hardy-Weinberg equilibrium p ≥ 0.001) and multivariable-adjusted residuals. We pursued a three-stage replication strategy of the GEE association results with 287 SNPs (P < 0.001 in Stage I) tested in Stage II (n ~1450 individuals) and 40 SNPs (P < 0.001 in joint analysis of Stages I and II) tested in Stage III (n~6650 individuals).

**Results:**

Long-term averages of LDL-C, HDL-C, and TG were highly heritable (h^2 ^= 0.66, 0.69, 0.58, respectively; each P < 0.0001). Of 70,987 tests for each of the phenotypes, two SNPs had p < 10^-5 ^in GEE results for LDL-C, four for HDL-C, and one for TG. For each multivariable-adjusted phenotype, the number of SNPs with association p < 10^-4 ^ranged from 13 to 18 and with p < 10^-3^, from 94 to 149. Some results confirmed previously reported associations with candidate genes including variation in the lipoprotein lipase gene (*LPL*) and HDL-C and TG (rs7007797; P = 0.0005 for HDL-C and 0.002 for TG). The full set of GEE, FBAT and linkage results are posted at the **d**ata**b**ase of **G**enotype **a**nd **P**henotype (dbGaP). After three stages of replication, there was no convincing statistical evidence for association (i.e., combined P < 10^-5 ^across all three stages) between any of the tested SNPs and lipid phenotypes.

**Conclusion:**

Using a 100K genome-wide scan, we have generated a set of putative associations for common sequence variants and lipid phenotypes. Validation of selected hypotheses in additional samples did not identify any new loci underlying variability in blood lipids. Lack of replication may be due to inadequate statistical power to detect modest quantitative trait locus effects (i.e., <1% of trait variance explained) or reduced genomic coverage of the 100K array. GWAS in FHS using a denser genome-wide genotyping platform and a better-powered replication strategy may identify novel loci underlying blood lipids.

## Introduction

Blood lipid levels are a major contributor to atherosclerotic cardiovascular disease [1]. Current evidence suggests that blood lipids are complex genetic phenotypes, influenced by both environmental and genetic factors. Heritability estimates for blood lipids are high, including ~40–60% for high-density lipoprotein cholesterol (HDL-C), ~40–50% for low-density lipoprotein cholesterol (LDL-C), and ~35–48% for triglycerides (TG) [2]. These estimates indicate that DNA sequence variation plays an important role in explaining inter-individual variation in blood lipid levels. Indeed, sequence variants in individual genes have been consistently related to blood lipid phenotypes, including *APOE/PCSK9 *with LDL-C [3-5], *CETP/LIPC/LPL *with HDL-C [6-9], and *APOA5/LPL *with TG [10,11], among others. However, the extent to which common genetic variants across the genome account for total variation in blood lipid levels is unknown.

Recent advances in genomics enable a genome-wide association study (GWAS), an approach in which a substantial fraction of common genetic variation is tested for a role in determining phenotypic variation [12]. These advances include a map of the correlation structure for approximately 4 million common genetic variants (minor allele frequency >5%) and whole-genome genotyping technologies capable of assaying 100,000–500,000 single nucleotide polymorphisms (SNPs) in an individual [13]. Utilizing a fixed genotyping marker set such as the Affymetrix 100K GeneChip in an association study tests a substantial fraction of the genome in whites, ~30–45% in some estimates [14]. GWAS has been successfully applied to identify novel genetic loci related to several medical phenotypes including age-related macular degeneration [15], inflammatory bowel disease [16], and electrocardiographic QT interval [17]. Identifying novel genetic variants related to blood lipid phenotypes may provide new drug targets to alter blood lipid levels and may aid in the prediction of cardiovascular disease.

We hypothesized that common genetic variants explain a proportion of the inter-individual variability in LDL-C, HDL-C, and TG. Accordingly, we conducted genome-wide linkage and association studies for these three phenotypes in Framingham Heart Study (FHS) participants.

## Materials and methods

### GWAS sample

Of the 1345 FHS participants who are part of the family plate set (see Executive Summary), we focused our analyses on the 1087 participants from the Offspring cohort who had Affymetrix 100K genotypes. Lipid phenotypes were measured at various examinations as described in Table 1. Each study participant provided written informed consent for genetic analyses and the study was approved by Boston University's Institutional Review Board.

**Table 1:** Lipid Phenotypes Examined Using Affymetrix 100K GeneChip Scan

Phenotype	Acronym	Phenotypes	N	h^2 ^*	Offspring Exam	Adjustment^† ^Multivariable model
Total cholesterol	TC	7	1069	0.57^‡^	1,2,3,4,5,6,7	Age, age^2^, smoking, body mass index, alcohol consumption, menopausal status, hormone replacement therapy; Covariate-adjusted residuals created separately by gender
Low-density lipoprotein cholesterol	LDL-C	7	1056	0.59^‡^	1,2,3,4,5,6,7	
High-density lipoprotein cholesterol	HDL-C	7	1062	0.52^‡^	1,2,3,4,5,6,7	
Triglycerides	TG	7	1068	0.48^‡^	1,2,3,4,5,6,7	
Mean total cholesterol	MeanTC	1	1087	0.61	Avg of Exams 1,2,3,4,5,6,7	
Mean low-density lipoprotein cholesterol	MeanLDL-C	1	1086	0.66	Avg of Exams 1,2,3,4,5,6,7	
Mean high-density lipoprotein cholesterol	MeanHDL-C	1	1087	0.69	Avg of Exams 1,2,3,4,5,6,7	
Mean triglycerides	MeanTG	1	1087	0.58	Avg of Exams 1,2,3,4,5,6,7	
Apolipoprotein A-I	ApoA-I	1	997	0.42	4	
Apolipoprotein B	ApoB	1	997	0.47	4	
Apolipoprotein C3	ApoC3	1	767	0.38	4	
Apolipoprotein E	ApoE	1	744	0.54	5	
High-density lipoprotein 2 cholesterol	HDL2-C	2	955 (exam 4) 958 (exam 5)	0.50 0.53	4,5	
High-density lipoprotein 3 cholesterol	HDL3-C	2	984 (exam 4) 1020 (exam 5)	0.48 0.54	4,5	
Large high-density lipoprotein by NMR	HDLNMRlg	1	851	0.53	4	
Intermediate high-density lipoprotein by NMR	HDLNMRint	1	851	0.22	4	
Small high-density lipoprotein by NMR	HDLNMRsm	1	851	0.24	4	
High-density lipoprotein particle size by NMR	HDLNMRsz	1	851	0.50	4	
Intermediate-density lipoprotein particle by NMR	IDLNMR	1	851	0.20	4	
Large low-density lipoprotein by NMR	LDLNMRlg	1	851	0.39	4	
Small low-density lipoprotein by NMR	LDLNMRsm	1	851	0.40	4	
Low-density lipoprotein particle size by NMR	LDLNMRsz	1	851	0.40	4	
Large very low-density lipoprotein particle by NMR	VLDLNMRlg	1	851	0.34	4	
Intermediate very low-density lipoprotein particle by NMR	VLDLNMRint	1	878	0.40	4	
Small very low-density lipoprotein particle by NMR	VLDLNMRsm	1	851	0.24	4	
Very low-density lipoprotein particle size by NMR	VLDLNMRsz	1	851	0.42	4	
Triglyceride/high-density lipoprotein cholesterol ratio	tghdl	7	1060	0.56^‡^	1,2,3,4,5,6,7	
Total cholesterol/high-density lipoprotein cholesterol ratio	cholhdl	7	1060	0.58^‡^	1,2,3,4,5,6,7	
Lipoprotein(a)	Lp(a)	1	763	0.90	3	
Remnant lipoprotein cholesterol	RLP-C	1	746	0.34	4	
Remnant lipoprotein triglycerides	RLP-TG	1	715	0.33	4	

Note: TG, MeanTG, cholhdl, and tghdl were natural log transformed due to skewed distribution;

*Heritability (h^2^) estimates presented are those after multivariable-adjustment; P < 0.0001 for all heritability estimates.

^†^Each phenotype had 2 adjustment schemes: age- and sex-adjusted and multivariable-adjusted. Both age- and sex-adjusted and multivariable-adjusted model results are web posted.

^‡^Heritability estimates were derived from lipid phenotypes at a single time-point, that of FHS Examination 1.

### Phenotype definition and methods

Blood lipids were measured from fasting venous blood collected at each of seven clinical examination time points extending from 1971 to 2001. Total cholesterol, HDL-C, and TG were measured by standard enzymatic methods. LDL-C was calculated using the Friedewald formula, with a missing value assigned for participants with a measured TG > 400 mg/dL. Clinical covariates utilized in phenotypic regression modeling included age at the time of blood lipid measurement, age^2^, body mass index (weight in kg divided by the height in m^2^), alcohol intake (drinks per week), current cigarette smoking (yes, no), menopausal status (postmenopausal yes, no), and hormone replacement therapy (yes, no).

Commonly-used lipid lowering therapies affect total cholesterol and TG. To account for treatment effect, we imputed total cholesterol and TG values for those treated with lipid-lowering therapy. The imputation procedure was modeled after prior work on imputing blood pressure values for those on antihypertensive medication [18]. For each treated individual, a correction factor was added to the observed [treated] lipid value (total cholesterol or TG). This correction factor consisted of the difference between an ''expected'' residual and the ''calculated'' residual. The ''calculated'' residual for each individual was generated in a sex-specific manner after adjustment for age, age^2^, age^3^, and examination year (by decade). The ''expected'' residual was generated within each sex and 10 year-age-group as the average of ''calculated'' residuals equal or greater than the treated individual's ''calculated'' residual.

Lipoprotein subclass profiles were measured by a commercially available proton NMR spectroscopic assay (LipoScience, Raleigh, NC) on plasma samples stored at -70°C as described previously [19]. The particle concentration of the following 9 lipoprotein species were determined: 3 VLDL subclasses [large, >60 nm (including chylomicrons); intermediate, 35–60 nm; small, 27–35 nm]; 3 LDL subclasses (IDL, 23–27 nm; large LDL 21.3–23 nm; small LDL, 18.3–21.2 nm); and 3 HDL subclasses (large, 8.8–13 nm; intermediate, 8.2–8.8 nm; small, 7.3–8.2 nm). The small LDL subclass comprises the sum of subclasses formerly labeled "intermediate" (19.8–21.2 nm) and "small" (18.3–19.7 nm) [19], since concentrations of both have very similar relations to lipid levels.

### Genotyping methods

All analyses were based on the Affymetrix 100K GeneChip genotyping data generated in Framingham Heart Study participants as described previously [20]. In order to minimize false positive associations due to genotyping artifact, we limited our analyses to SNPs with a genotyping call rate ≥80% and a Hardy-Weinberg Equilibrium P ≥ 0.001. Given lower statistical power to detect associations with rarer SNPs, we limited our results to SNPs with a minor allele frequency ≥10%.

### Statistical analysis methods

TG levels were log-transformed to approximate a normal distribution. For each blood lipid phenotype, the long-term average of 4 to 7 serial measurements was used as the primary phenotype. Participants contributing fewer than 4 of 7 measures of a given phenotype were excluded from that analysis. MeanLDL-C, MeanHDL-C, and MeanTG were adjusted for covariates in sex-specific linear regression models. Two sets of phenotypic models were created: Model 1 (age, age^2^) and Model 2 (age, age^2^, body mass index, alcohol intake, cigarette smoking, menopausal status, and hormone replacement therapy). For quantitative covariates (age, body mass index, and alcohol intake), the mean value across examinations was used as a covariate. For categorical covariates, the proportion of exams scored as 'yes' was used. The residual MeanLDL-C, MeanHDL-C, and MeanlogTG values from Model 1 and Model 2 served as the primary phenotypes.

For genotype-phenotype association analyses, we assumed an additive model of inheritance. We conducted multivariable linear regression using GEE, family-based association testing using FBAT, and linkage using Merlin for computation of IBDs and SOLAR for variance component models as described in the Executive Summary.

### Heritability analyses

Heritability estimates for the lipid phenotypes were obtained from extended families with at least two members by variance-components methods using the Sequential Oligogenic Linkage Analysis Routines (SOLAR) package [21]. Using this approach, maximum-likelihood estimation was applied to a mixed-effects model that incorporated fixed covariate effects, additive genetic effects, and residual error. The additive genetic effects and residual errors were assumed to be normally distributed and to be mutually independent. The analyses were performed using residuals from the multivariable models (Model 1 and Model 2) mentioned above. For phenotypes with kurtosis > 1, heritability estimates were computed on ranked normalized deviates.

### Replication samples

Replication genotyping was attempted in three independent sample sets: a) the FHS unrelated plate set; b) Genetics of Lipid Lowering Drugs and Diet Network (GOLDN); and c) Malmö Diet and Cancer Study – Cardiovascular Cohort (MDC-CC).

The second stage consisted of ~1450 biologically unrelated individuals from the FHS unrelated plate set. The third stage consisted of ~1450 participants from GOLDN and ~5200 participants from MDC-CC. GOLDN is a family-based sample recruited from two National Heart, Lung, and Blood Institute's Family Heart Study field centers (Minneapolis, MN and Salt Lake City, UT). The Family Heart Study is a multi-center, population-based cohort designed to study the genetic and environmental determinations of cardiovascular disease.

The MDC study is a community-based prospective epidemiologic cohort of 28,098 persons recruited for a baseline examination between 1991 and 1996. From this cohort, 6103 persons were randomly selected to participate in the MDC-CC which sought to investigate risk factors for cardiovascular disease. Of the MDC-CC participants, 5466 had DNA and lipid phenotypes available. Individuals on lipid lowering therapy and with outlier values of LDL-C, HDL-C, or TG (top 0.5% of the distribution) were excluded, leaving 5212 individuals available for the SNP-lipid association analyses

### Staged replication strategy

For follow-up into Stage II (the FHS unrelated plate set), we selected all SNPs in the GWAS with an association P < 0.001 for the MeanLDL-C, MeanHDL-C, or MeanTG phenotypes from the minimally-adjusted phenotypic model (Model 1, adjustment for age, age^2 ^only). We next conducted a joint analysis of Stage I (GWAS 100K data) and Stage II (FHS unrelated plate set). The joint analysis consisted of a weighted average of the beta estimates and standard errors from Stages I and II and used the inverse of the variance in each stage as weights.

For follow-up into Stage III (GOLDN and MDC-CC), we selected for genotyping all SNPs with a P < 0.001 in the joint analysis of Stages I and II. For genotype-phenotype association analyses in MDC-CC and GOLDN, we assumed an additive model of inheritance. In MDC-CC, we conducted multivariable linear regression analyses to test the null hypothesis that LDL-C, HDL-C, or TG residuals (sex-specific residuals adjusted for age and age^2^) did not differ by increasing minor allele copy number. In GOLDN, to account for correlated observations due to family relationships we used linear mixed-effects methods in SOLAR.

To summarize the statistical evidence for association for each SNP across all three stages, we reiterated the weighted average beta-estimates and standard errors as described above.

## Results

Clinical characteristics of the FHS sample of 1345 subjects are presented in the Executive Summary. Table 1 displays the variables that were studied in our analyses of lipid phenotypes. Further information on these phenotypes can be found at http://www.ncbi.nlm.nih.gov/projects/gap/cgi-bin/study.cgi?id=phs000007. Since Original cohort members were non-fasting at examination, our analyses considered only the 1087 Offspring Study participants with fasting lipid measurements and Affymetrix 100K SNP genotypes. For this paper we focus on longitudinal mean levels of serially measured values (minimum of 4, maximum of 7) of LDL-C, HDL-C, and TG (labeled MeanLDL-C, MeanHDL-C, and MeanTG).

Heritability estimates for long-term average lipid phenotypes (Mean LDL-C, MeanHDL-C, and MeanTG) were greater than those from single time-point measurements (Table 1). For example, the heritabilities of MeanLDL-C, MeanHDL-C, and MeanTG were 0.66, 0.69, and 0.58, respectively, whereas heritabilities for LDL-C, HDL-C, and TG measured at FHS Examination 1 (a single time-point) were 0.59, 0.52, and 0.48, respectively. The highest heritability estimate for any available lipid phenotype was that for lipoprotein (a) at 0.90.

From the GEE analyses, the strongest associations for MeanLDL-C, MeanHDL-C, and MeanTG were for SNPs rs287474 (p = 6.3*10^-9^), rs524802 (p = 7.6*10^-7^), and rs7007075 (p = 7.7*10^-6^), respectively (Table 2a). From the FBAT analyses, the strongest associations for MeanLDL-C, MeanHDL-C, and MeanTG were for SNPs rs287474 (p = 1.4*10^-8^), rs10495594 (p = 5.1*10^-5^), and rs1449866 (p = 1.8*10^-5^), respectively (Table 2b). For each multivariable-adjusted phenotype, the number of SNPs with a GEE association p < 10^-4 ^ranged from 13 to 18 and with p < 10^-3^, from 94 to 149. The number of SNPs with FBAT association p < 10^-4 ^ranged from 2 to 5 and with p < 10^-3^, from 74 to 79.

**Table 2:** Overview of Top Association and Linkage Results for MeanLDL-C, MeanHDL-C, and MeanTG

2a. Top 25 SNPs for association with MeanLDL-C, MeanHDL-C, or MeanTG based on the lowest p values of the GEE tests						
**Phenotype**	SNP rs ID*	**Chr**	**Physical location (bp)**	**GEE P-value**	**FBAT P-value**	**gene (IN or NEAR)**

MeanLDL-C	rs287474	13	68173961	6.3*10^-9^	1.4*10^-8^	
MeanLDL-C	rs287354	13	68137953	3.4*10^-8^	6.8*10^-8^	
MeanHDL-C	rs524802	19	42138787	7.6*10^-7^	0.04	
MeanHDL-C	rs505717	19	42122156	1.2*10^-6^	0.07	ZNF345
MeanHDL-C	rs544543	19	42122663	2.5*10^-6^	0.07	
MeanTG	rs7007075	8	138168448	7.7*10^-6^	0.004	
MeanHDL-C	rs3734678	6	107639853	8.2*10^-6^	0.009	C6orf210
MeanLDL-C	rs10488824	11	81678287	1.4*10^-5^	0.03	
MeanTG	rs2142136	10	89753050	1.5*10^-5^	0.06	BC005821| PTEN
MeanLDL-C	rs1555173	6	85121177	1.7*10^-5^	0.09	
MeanHDL-C	rs688933	9	70902713	1.8*10^-5^	0.38	TRPM3| AJ505026| AL136545
MeanTG	rs314474	11	11482634	1.8*10^-5^	0.09	AB094146| IGSF4| BC047021
MeanHDL-C	rs966376	18	27086836	2.1*10^-5^	0.07	
MeanHDL-C	rs4459845	3	127400326	2.3*10^-5^	0.005	ALDH1L1| CR749807
MeanTG	rs220840	11	114826173	2.5*10^-5^	0.09	AB094146| IGSF4| BC047021
MeanHDL-C	rs3850301	14	58150885	2.8*10^-5^	8.6*10^-4^	BX161433| DACT1
MeanLDL-C	rs1317538	6	73706132	3.0*10^-5^	0.02	BC050689| KCNQ5
MeanHDL-C	rs7021834	9	71123639	3.0*10^-5^	0.003	AL136545
MeanLDL-C	rs504527	1	34142701	3.2*10^-5^	0.007	CSMD2
MeanHDL-C	rs10488779	11	79494759	3.6*10^-5^	0.07	
MeanLDL-C	rs2415621	14	40114277	3.8*10^-5^	0.005	
MeanHDL-C	rs10508518	10	17081479	3.9*10^-5^	0.09	CUBN
MeanLDL-C	rs1245058	1	69760085	4.1*10^-5^	0.004	LRRC7
MeanHDL-C	rs10488780	11	79490000	4.4*10^-5^	0.06	
MeanHDL-C	rs541326	9	70936505	4.5*10^-5^	0.39	TRPM3| AJ505026| AL136545

**2b. Top 25 SNPs for association with MeanLDL-C, MeanHDL-C, or MeanTG based on the lowest p values of the FBAT tests**						

**Phenotype**	SNP rs ID*	**Chr**	**Physical location (bp)**	**GEE P-value**	**FBAT P-value**	**gene (IN or NEAR)**

MeanLDL-C	rs287474	13	68173961	1.4*10^-8^	6.3*10^-9^	
MeanLDL-C	rs287354	13	68137953	6.8*10^-8^	3.4*10^-8^	
MeanTG	rs1449866	3	144359331	1.8*10^-5^	0.002	CHST2| AK131346
MeanTG	rs10506354	12	57759034	2.2*10^-5^	0.007	
MeanTG	rs10494989	1	211576634	2.4*10^-5^	4.7*10^-4^	AY552981| AK123409| KCNK2
MeanTG	rs2371978	12	57759239	2.4*10^-5^	0.01	
MeanHDL-C	rs10495594	2	12280997	5.1*10^-5^	1.8*10^-4^	
MeanHDL-C	rs10495593	2	12279945	5.1*10^-5^	1.8*10^-4^	
MeanLDL-C	rs1411931	1	37451967	5.8*10^-5^	0.03	
MeanLDL-C	rs10516433	4	99800704	7.7*10^-5^	0.03	TSPAN5
MeanHDL-C	rs2182114	1	111496269	8.2*10^-5^	0.07	CHI3L2
MeanTG	rs1835353	2	124880057	8.3*10^-5^	0.02	CNTNAP5| AK056528
MeanLDL-C	rs10516434	4	99802493	8.6*10^-5^	0.03	TSPAN5
MeanLDL-C	rs10507755	13	68355991	1.1*10^-4^	1.6*10^-4^	
MeanLDL-C	rs6853079	4	99800789	1.3*10^-4^	0.06	TSPAN5
MeanHDL-C	rs1508116	2	41437773	1.3*10^-4^	0.07	
MeanLDL-C	rs10518072	4	71271418	1.4*10^-4^	0.05	APIN| C4orf7| CSN3
MeanTG	rs715260	4	9804415	1.5*10^-4^	0.002	WDR1
MeanTG	rs7784056	7	77762038	1.6*10^-4^	0.12	AB014605| MAGI2
MeanHDL-C	rs6505623	18	1115057	1.6*10^-4^	0.006	
MeanHDL-C	rs1541296	18	59173095	1.6*10^-4^	0.02	FVT1
MeanHDL-C	rs6821328	4	54058427	1.7*10^-4^	0.02	SCFD2
MeanTG	rs1501572	9	2894794	1.7*10^-4^	1.1*10^-4^	
MeanTG	rs4684343	3	2366098	1.8*10^-4^	0.24	CNTN4
MeanTG	rs225634	6	142518312	1.9*10^-4^	2.4*10^-4^	C6orf55

**2c. Magnitude and Location of Peak LOD scores > 2.0 for MeanLDL-C, MeanHDL-C, and MeanTG**						

**Phenotype**	**Exam**	**Chr**	**Physical location (Mb)**	**Maximum LOD**	**LOD-1.5 Interval**	**LOD+1.5 Interval**

MeanHDL-C	1–7	7	33485983	3.30	27810820	36074331
MeanLDL-C	1–7	9	94130181	2.83	88057135	98516033
MeanTG	1–7	1	153444389	2.73	151582274	155505440
MeanLDL-C	1–7	3	196384998	2.12	187706181	199138789

Chr = chromosome

Linkage LOD scores > 2.0 are presented in Table 2c. The best evidence for linkage was a peak LOD score of 3.3 on chromosome 7 for the MeanHDL-C phenotype.

Because the prior probability of any SNP relating to a phenotype is low and given the number of tests, the P value distribution in a GWAS should approach a null distribution. Any strong departure from this expectation might suggest artifacts in genotyping or analysis. For the 70,987 SNPs that passed quality-control filters, the distribution of association P values (generated by the GEE methodology) approached a null distribution but with a slight excess of low P values. For example, for the MeanLDL-C, whereas one would expect 1% of SNPs to demonstrate a P < 0.01 by chance, we found that 1.34% of SNPs displayed a P < 0.01. Similar results were seen for meanHDL-C and meanTG (data not shown).

We evaluated the association results for a SNP and each of a set of four correlated phenotypes – ApoA-I, LDLNMRsm, MeanHDL-C, and MeanTG (Table 3). Several SNPs were associated with P < 0.01 for 3 of the 4 phenotypes.

**Table 3:** GEE results for 4 correlated phenotypes (ApoA-I, LDLNMRsm, MeanHDL-C, and MeanTG), ranked by proportion of GEE P < 0.01*

rs id	Chr	Physical Position	GEE P ApoA-I	GEE P LDLNMRsm	GEE P MeanHDL-C	GEE P MeanTG	Gene Symbol
rs505717	19	42122156	7.8*10^-4^	0.003	1.3*10^-6^	0.04	ZNF568
rs3734678	6	107639853	4.7*10^-4^	0.18	8.2*10^-6^	0.006	PDSS2
rs2256038	21	15443047	0.11	0.002	8.8*10^-4^	6.4*10^-5^	
rs10486788	7	15788581	4.7*10^-4^	0.005	8.5*10^-4^	0.01	
rs232885	1	58824078	0.86	0.002	9.6*10^-4^	5.7*10^-4^	AB067502
rs4982795	14	23294875	0.001	1.9*10^-7^	0.001	0.11	
rs524802	19	42138787	0.001	0.003	7.6*10^-7^	0.02	ZNF568
rs544543	19	42122663	0.001	0.004	2.5*10^-4^	0.03	ZNF568
rs2823097	21	15445015	0.10	0.004	0.002	8.0*10^-5^	
rs1394041	3	148579545	0.02	0.003	0.002	5.6*10^-5^	
rs10499320	7	2906624	0.001	0.001	8.8*10^-4^	0.03	
rs2889195	1	65868751	6.6*10^-4^	0.003	0.003	0.02	
rs7007797	8	19921250	0.01	0.02	5.0*10^-4^	0.002	
rs7597861	2	100311705	0.08	0.004	1.0*10^-4^	0.006	
rs6063858	20	50715959	0.04	3.0*10^-4^	0.002	0.003	
rs2425524	20	40750358	0.01	0.05	3.6*10^-4^	0.008	
rs1509384	2	22556488	8.5*10^-4^	0.01	0.007	0.03	
rs9317760	13	68480686	0.003	0.05	6.6*10^-4^	0.002	
rs6804032	3	116031161	0.002	0.02	0.009	8.7*10^-4^	
rs8069913	17	13812167	0.03	0.009	0.001	6.7*10^-4^	

*All SNPs in this table had GEE association P values < 0.01 for at least three of the four traits (ApoA-I, LDLNMRsm, MeanHDL-C, and MeanTG). No SNPs in our dataset had GEE association P values < 0.01 for all four traits.

Among the GEE association results, a SNP (rs7007797) in the lipoprotein lipase (LPL) was associated with MeanHDL-C (p = 0.0005) and MeanTG (p = 0.002) (Table 4). This SNP is a perfect proxy (r^2 ^= 1) to the previously studied rs328 (also known as S447X) [22]. The minor allele of rs328 has been consistently related to higher HDL-C and lower TG. The direction of effect for SNP rs7007797 in our dataset was consistent with previous observations. Due to a lack of SNPs in the Affymetrix 100K GeneChip correlated with previously reported variants (at r^2 ^> 0.5 threshold) in the *APOE*, *PCSK9*, *CETP*, *LIPC*, and *APOA5 *genes, we were unable to confirm these other previously reported associations (Table 4).

**Table 4:** Comparison with the prior literature

Gene	Phenotype	Selected SNPs previously associated with phenotype	# SNPs in Affymetrix 100K within 60 kb of gene	# SNPs in Affymetrix 100K within 60 kb of Gene Locus and with r^2 ^> 0.5 to previously reported SNP	SNP in Affymetrix 100K (r^2 ^to previously reported SNP)	p for association in FHS 100K
*APOE*	LDL-C	rs429358 rs7412	1	0	-	-
*PCSK9*	LDL-C	rs11591147	7	0	-	-
*CETP*	HDL-C	rs1800775	2	0	-	-
*LIPC*	HDL-C	rs1800588	18	0	-	-
*LPL*	HDL-C	rs328	8	2	rs10503669 (r^2 ^= 1.0) rs7007797 (r^2 ^= 1.0)	rs10503669 – 9.1*10^-4^ rs7007797 – 5.0*10^-4^
*LPL*	TG	rs328	8	2	rs10503669 (r^2 ^= 1.0) rs7007797 (r^2 ^= 1.0)	rs10503669 – 0.02 rs7007797 – 0.002
*APOA5*	TG	rs662799 rs3135506	4	0	-	-

Replication is critical to distinguish true positives from false ones in a GWAS. We pursued a three-stage replication strategy with 287 SNPs (P < 0.001 in Stage I) tested in Stage II (n~1450 individuals) and 40 SNPs (P < 0.001 in joint analysis of Stages I and II) tested in Stage III (n~6650 individuals). Results are displayed in Table 5. After three stages of replication, there was no convincing statistical evidence for association (i.e. joint analysis stages I, II & III P < 10^-5^) between any of the tested SNPs and lipid phenotypes.

**Table 5:** Association Results for 40 SNPs Attempted for Replication in Three Stages

SNP	Chr	Gene	Allele*	Trait	Stage I – FHS 100K			Stage II – FHS Unrelated			Joint P	Stage III – MDC			Stage III – GOLDN			Joint Stages I, II, & III		
					**P**	**Beta^†^**	**SE**	**P**	**Beta**	**SE**	**Stages I & II**	**P**	**Beta**	**SE**	**P**	**Beta**	**SE**	**P**	**Beta**	**SE**

rs7231460	18		C	MeanHDL-C	4.3*10^-4^	-0.145	0.041	2.4*10^-4^	-0.131	0.036	3.7*10^-7^	0.50	0.013	0.020	0.60	0.023	0.044	0.03	-0.033	0.015
rs966376	18		C	MeanHDL-C	2.5*10^-4^	-0.148	0.040	4.8*10^-4^	-0.125	0.036	4.5*10^-7^	0.43	0.016	0.020	0.56	0.026	0.044	0.04	-0.031	0.015
rs744134	13	L10374	C	MeanLDL-C	5.4*10^-4^	0.168	0.048	4.7*10^-4^	0.124	0.036	1.1*10^-6^	0.67	0.009	0.020	0.20	0.062	0.048	0.0007	0.053	0.016
rs7233386	18		T	MeanHDL-C	2.4*10^-4^	0.149	0.041	0.005	0.100	0.036	6.3*10^-6^	0.32	-0.020	0.020	0.61	-0.023	0.046	0.11	0.024	0.015
rs1428445	5		A	MeanHDL-C	3.0*10^-4^	-0.187	0.052	0.01	-0.113	0.044	1.8*10^-5^	0.24	-0.028	0.024	0.77	0.016	0.054	0.002	-0.058	0.018
rs2304589	2	LOC130502	A	MeanLDL-C	6.4*10^-4^	0.187	0.055	0.006	0.124	0.045	1.8*10^-5^	0.47	-0.020	0.027	0.09	-0.101	0.060	0.18	0.027	0.020
rs2278528	2	LOC130502	G	MeanLDL-C	6.7*10^-4^	0.180	0.053	0.007	0.122	0.045	2.0*10^-5^	0.51	-0.018	0.027	0.12	-0.092	0.060	0.14	0.029	0.020
rs2142136	10	PTEN	G	MeanTG	8.1*10^-6^	0.228	0.051	0.14	0.077	0.052	2.4*10^-5^	0.38	-0.026	0.029	0.85	0.011	0.061	0.06	0.040	0.021
rs1555173	6		C	MeanLDL-C	1.0*10^-5^	-0.243	0.055	0.10	-0.083	0.050	2.7*10^-5^	0.31	-0.027	0.027	0.03	0.143	0.066	0.01	-0.051	0.021
rs6053754	20	FLJ25067	C	MeanLDL-C	3.1*10^-4^	0.166	0.046	0.02	0.090	0.037	3.4*10^-5^	0.46	0.017	0.022	0.99	-0.001	0.050	0.003	0.049	0.017
rs8032553	15	KIF7	A	MeanLDL-C	3.0*10^-5^	0.171	0.041	0.08	0.061	0.034	5.5*10^-5^	0.09	0.033	0.020	Failed	Failed	Failed	0.0002	0.060	0.016
rs1741662	6	RFXDC1	T	MeanTG	7.2*10^-5^	0.198	0.050	0.07	0.083	0.046	5.9*10^-5^	0.15	-0.037	0.026	0.03	-0.131	0.061	0.58	0.011	0.020
rs1741660	6	RFXDC1	T	MeanTG	7.8*10^-5^	0.197	0.050	0.07	0.084	0.046	6.1*10^-5^	Failed^‡^	Failed	Failed	Failed	Failed	Failed	6E-05	0.136	0.034
rs890945	5		A	MeanHDL-C	5.4*10^-4^	-0.181	0.052	0.03	-0.099	0.044	8.0*10^-5^	0.18	-0.033	0.024	0.79	0.014	0.053	0.002	-0.057	0.018
rs10512297	9		T	MeanLDL-C	2.1*10^-4^	-0.235	0.064	0.05	-0.108	0.054	8.6*10^-5^	0.71	-0.011	0.030	0.44	0.056	0.072	0.03	-0.051	0.023
rs541326	9	TRPM3	G	MeanHDL-C	3.6*10^-5^	-0.201	0.049	0.28	-0.061	0.056	1.3*10^-4^	0.10	0.048	0.029	0.02	-0.159	0.069	0.08	-0.038	0.022
rs719829	5		G	MeanTG	1.3*10^-4^	-0.156	0.041	0.11	-0.062	0.039	1.4*10^-4^	0.66	0.009	0.022	0.58	0.028	0.050	0.08	-0.028	0.016
rs10509384	10	KCNMA1	G	MeanLDL-C	7.7*10^-4^	-0.148	0.044	0.04	-0.076	0.038	1.9*10^-4^	0.02	0.050	0.021	Failed	Failed	Failed	0.79	-0.005	0.017
rs688933	9	TRPM3	T	MeanHDL-C	3.1*10^-6^	-0.232	0.050	0.80	-0.015	0.059	2.0*10^-4^	0.17	0.041	0.030	0.01	-0.172	0.069	0.05	-0.044	0.022
rs1122080	5		A	MeanHDL-C	3.4*10^-4^	-0.202	0.056	0.07	-0.084	0.047	2.5*10^-4^	0.44	-0.020	0.025	0.47	-0.041	0.058	0.005	-0.055	0.019
rs6743779	2	KLF7	C	MeanLDL-C	2.4*10^-5^	-0.181	0.043	0.24	-0.043	0.036	2.8*10^-4^	0.69	0.009	0.022	Failed	Failed	Failed	0.05	-0.033	0.017
rs950828	4		A	MeanHDL-C	9.1*10^-4^	-0.151	0.046	0.06	-0.080	0.043	2.9*10^-4^	0.58	0.012	0.022	Failed	Failed	Failed	0.10	-0.030	0.018
rs1365032	8		C	MeanLDL-C	1.4*10^-4^	-0.161	0.042	0.12	-0.053	0.034	3.2*10^-4^	0.01	-0.056	0.020	0.11	0.077	0.048	0.0002	-0.055	0.015
rs287474	13		T	MeanLDL-C	8.1*10^-6^	0.209	0.047	0.16	0.056	0.040	3.4*10^-4^	0.97	0.001	0.020	0.62	-0.023	0.046	0.05	0.030	0.016
rs10507756	13		C	MeanLDL-C	6.0*10^-5^	0.202	0.050	0.22	0.052	0.043	4.2*10^-4^	0.24	0.032	0.028	0.89	-0.008	0.060	0.003	0.059	0.020
rs1507203	13		C	MeanLDL-C	1.9*10^-4^	0.172	0.046	0.15	0.057	0.039	4.3*10^-4^	0.23	0.027	0.023	0.32	-0.052	0.052	0.01	0.044	0.017
rs4941236	18	SERPINB8	C	MeanLDL-C	4.7*10^-4^	0.151	0.043	0.09	0.059	0.034	4.3*10^-4^	0.83	-0.004	0.020	0.18	-0.065	0.048	0.02	0.022	0.015
rs544543	19		G	MeanHDL-C	4.8*10^-6^	-0.185	0.040	0.61	-0.019	0.038	4.7*10^-4^	0.95	-0.001	0.021	0.78	-0.013	0.047	0.03	-0.033	0.016
rs3763188	6	REPS1	C	MeanTG	5.2*10^-4^	-0.191	0.055	0.12	-0.076	0.048	5.1*10^-4^	0.13	0.042	0.028	0.02	-0.153	0.063	0.09	-0.035	0.021
rs717987	6	RUNX2	G	MeanHDL-C	8.0*10^-5^	0.250	0.063	0.26	0.063	0.057	5.4*10^-4^	0.06	0.058	0.031	0.87	0.011	0.067	0.0007	0.079	0.023
rs10508997	10		G	MeanTG	4.1*10^-4^	-0.165	0.047	0.15	-0.062	0.043	5.6*10^-4^	0.10	0.039	0.023	Failed	Failed	Failed	0.51	-0.012	0.019
rs2395833	7	PRKAR2B	T	MeanHDL-C	6.2*10^-4^	-0.145	0.042	0.12	-0.060	0.038	5.6*10^-4^	0.99	0.000	0.021	0.06	-0.090	0.049	0.01	-0.041	0.016
rs6677589	1	JUN	G	MeanTG	1.9*10^-4^	-0.145	0.039	0.27	-0.042	0.037	7.1*10^-4^	0.18	0.028	0.021	0.84	-0.010	0.048	0.30	-0.016	0.016
rs10513320	3		A	MeanTG	1.3*10^-4^	-0.190	0.050	0.31	-0.046	0.045	9.0*10^-4^	0.24	0.028	0.024	0.21	0.068	0.054	0.61	-0.010	0.018
rs508969	18	CETN1	A	MeanLDL-C	6.8*10^-4^	-0.144	0.042	0.18	-0.053	0.040	9.5*10^-4^	0.31	0.023	0.023	0.64	0.025	0.054	0.29	-0.018	0.017
rs7858099	9		G	MeanLDL-C	6.9*10^-4^	-0.140	0.041	0.15	-0.051	0.035	9.6*10^-4^	0.53	-0.013	0.021	0.31	-0.049	0.048	0.006	-0.043	0.016
rs4134425	14		T	MeanLDL-C	6.6*10^-4^	-0.156	0.046	0.13	-0.055	0.036	9.8*10^-4^	0.14	0.032	0.021	0.16	-0.068	0.048	0.24	-0.019	0.016
rs7021834	9	AL136545	T	MeanHDL-C	4.3*10^-5^	-0.174	0.043	0.65	-0.021	0.045	9.9*10^-4^	0.05	0.045	0.023	1.00	0.000	0.057	0.72	-0.006	0.018
rs10500770	11		T	MeanLDL-C	3.9*10^-4^	-0.157	0.044	0.17	-0.049	0.036	0.001	0.41	0.017	0.021	0.90	0.006	0.050	0.22	-0.019	0.016
rs10516430	4	RAP1GDS1	T	MeanHDL-C	7.2*10^-4^	-0.139	0.041	0.20	-0.052	0.040	0.001	0.64	-0.010	0.022	Failed	Failed	Failed	0.02	-0.041	0.017

*The allele on the positive strand of the reference genome was modeled in all analyses.

^†^Beta refers to the proportion of 1 standard deviation unit change in phenotype (phenotype is sex-specific residual adjusted for age and age^2^) per copy of the allele modeled.

^‡^"Failed" refers to SNP genotype failure in the sample.

## Discussion

We examined associations of Affymetrix 100K SNPs and lipid traits in FHS and identified putative associations with lipid phenotypes. We studied the long-term average of up to 7 measurements each of LDL-C, HDL-C, and TG as the primary phenotypes and for one phenotype, the MeanLDL-C, we observed a nominal P that exceeded genome-wide significance [13]. However, validation of selected hypotheses in additional samples did not identify any new loci underlying variability in blood lipids.

GWAS offers the potential to identify novel genetic variants/loci that are associated with blood lipid variation, unlimited by our current knowledge of lipoprotein biology. However, a central limitation of GWAS is that the true signals are mixed amidst a large number of false positive results. Validation in additional samples is required to distinguish the true positives from the false ones.

Replication of initial GWAS findings using a staged design has been suggested to minimize genotyping cost and maximize statistical power [23,24]. An important consideration in such a design is the proportion of markers taken forward to a second stage. We estimated the statistical power for our three-stage GWAS strategy. Assuming a modest number of markers (all SNPs with P < 0.001 for each phenotype, ~0.1% of markers) are taken forward to Stage II, a second stage sample size of 1450, that SNPs with P < 0.001 are taken forward from Stage II to Stage III, a stage III sample size of 6650, and that the final alpha (after Stages I, II, & III) is set at a conservative 5*10^-8^, we estimated that we had 89% power to detect a quantitative trait locus explaining 2% of phenotypic variance, 48% power to detect a locus explaining 1% of the variance, and 13% power to detect a locus explaining 0.5% of the variance.

With our replication effort, we failed to identify any novel loci related to blood lipids. At least two potential explanations are possible. First, our study design had limited statistical power to detect common SNPs that explain ≤1% of trait variance. In the Diabetes Genetics Initiative genome-wide association study for blood lipid traits, we recently showed that for lipid traits, there are few common variants that explain >2% of the variance and most SNPs explain <1% of trait variance [25]. To have adequate statistical power to detect these effects given an initial GWAS sample size of ~1000, many more markers (i.e., hundreds of SNPs) will need to be taken to the second and third stages. Second, the limited genomic coverage of the Affymetrix 100K array may have limited our ability to replicate previously reported loci and discover novel loci. For example, using the Affymetrix 500 K array, we recently identified glucokinase regulatory protein (*GCKR*) as a novel locus associated with TG [25]. Of any SNP on the 500 K array, an intronic *GCKR *SNP (rs780094) explained the greatest proportion of blood TG variance in the Diabetes Genetics Initiative study. However, on the Affymetrix 100K array, there are no SNPs within the 60 kb spanning *GCKR*.

## Strengths and limitations

This study is distinguished by the availability of serial lipid phenotypes over a 30-year time span, the community-based nature of the collection, and the routine ascertainment of covariates in a standardized clinical examination. We acknowledge several limitations. These include the lack of validation for the imputation methodology used to address lipid lowering therapy, limited statistical power due to sample size, and confinement to a single ancestral group – whites of European ancestry.

## Conclusions & future directions

Using a 100K genome-wide scan, we present association and linkage results for a rich set of lipid phenotypes in FHS. This resource may be useful for comparisons with other GWAS currently in progress. GWAS in FHS using a denser genome-wide genotyping platform and a better-powered replication strategy may identify novel loci underlying blood lipids.

## Abbreviations

FBAT = family-based association test; GEE = generalized estimating equations.

## Competing interests

The authors declare that they have no competing interests.

## Authors' contributions

SK, AM, SD, GP, JMO, and LAC participated in the design of the study and the interpretation of the data. AM, GP, and SD conducted the statistical analyses. SK drafted the manuscript. AS, CG, LG, and NPB generated replication genotype data and analyses. OM and MOM provided replication samples and conducted association analyses in the Malmo Diet and Cancer Study. DKA and JMO provided replication samples and led the generation of lipid phenotypes in the GOLDN study. SD, SK, RD, JMO, and LAC revised the manuscript critically for important intellectual content. All authors read and approved the above manuscript.
